# In vitro digestion and colonic fermentation of phenolic compounds and their antioxidant potential in Australian beach-cast seaweeds

**DOI:** 10.1038/s41598-024-54312-5

**Published:** 2024-02-22

**Authors:** Vigasini Subbiah, Faezeh Ebrahimi, Osman Tuncay Agar, Frank R. Dunshea, Colin J. Barrow, Hafiz A. R. Suleria

**Affiliations:** 1 Centre for Sustainable Bioproducts, Deakin University, Waurn Ponds, VIC 3217 Australia; 2 School of Agriculture, Food and Ecosystem Sciences, Faculty of Science, The University of Melbourne, Parkville, VIC 3010 Australia

**Keywords:** Macroalgae, Bioactive compounds, Gastrointestinal digestion, Bioaccessibility, Short-chain fatty acids, Biochemistry, Chemical biology

## Abstract

Beach-cast seaweed has recently garnered attention for its nutrient-rich composition, including proteins, carbohydrates, vitamins, minerals, and phytochemicals. This study focuses on the phenolic content and antioxidant potential of five Australian beach-cast seaweed species during in vitro digestion and colonic fermentation. The bioaccessibility of the selected phenolic compounds was estimated and short chain fatty acids (SCFAs) production was determined. *Cystophora* sp., showed a notable increase in phenolic content (23.1 mg GAE/g) and antioxidant capacity (0.42 mg CE/g) during the intestinal and gastric phases of in vitro digestion. *Durvillaea* sp. demonstrated a significant release of flavonoids (0.35 mg QE/g), while *Phyllosphora comosa* released high levels of tannins (0.72 mg CE/g) during the intestinal phase. During colonic fermentation, *P. comosa* released the highest levels of phenolic compounds (4.3 mg GAE/g) after 2 h, followed by an increase in flavonoids (0.15 mg QE/g), tannins (0.07 mg CE/g), and antioxidant activity (DPPH: 0.12 mg TE/g; FRAP: 0.61 mg TE/g) after 4 h. Moreover, *P. comosa* released a considerable amount of phenolic compounds during both in vitro digestion and colonic fermentation. All species consistently released phenolic compounds throughout the study. Phloroglucinol, gallic acid, and protocatechuic acid were identified as the most bioaccessible phenolic compounds in all five Australian beach-cast seaweeds in the in vitro digestion. Nevertheless, compound levels declined during the colonic fermentation phase due to decomposition and fermentation by gut microbiota. With regard to SCFAs, *P. comosa* displayed elevated levels of acetic (0.51 mmol/L) and propionic acid (0.36 mmol/L) at 2 h, while *Durvillaea* sp. showed increased butyric (0.42 mmol/L) and valeric (0.26 mmol/L) production acid after 8 h. These findings suggest that seaweed such as *Cystophora* sp., *Durvillaea* sp., and *P.* comosa are promising candidates for food fortification or nutraceutical applications, given their rich phenolic content and antioxidant properties that potentially offer gut health benefits.

## Introduction

Seaweeds, including beach-cast seaweeds, are potential sources of bioactive components due to their diverse chemical composition, which includes proteins, carbohydrates, vitamins, minerals, and phytochemicals^1^. Among these, phenolic compounds such as phlorotannin, phenolic acids, and flavonoids are particularly noteworthy for their range of health-promoting properties, including antioxidant, neuro-regenerative, anti-inflammatory, and anticancer effects. Notable phenolic compounds identified in seaweeds include gallic acid, catechin, epicatechin, and others, which contribute to their high phenolic content and antioxidant properties^2–5^.

Seaweed phenolics, due to their significant health benefits, have garnered attention as potential bioactive compounds in human diets^1^. Upon consumption, seaweed phenolics are broken down and absorbed into the human digestive system^6^. In vitro digestion studies involve replicating the conditions of the stomach and small intestine using enzymes and other digestive fluids^7^. The phenolic content of brown seaweed *Ascophyllum nodosum* decreased after in vitro digestion, possibly due to the breakdown of these compounds by digestive enzymes^8^. However, Mercatante et al.^9^ showed an increase in the antioxidant potential of seaweed after the in vitro digestion, which was possibly due to the release of bound phenolic compounds during digestion. The released phenolic compounds become bioaccessible and so contributed to observed bioactiviy^10^.

Pigs (*Sus scrofa*) are considered optimal non-primate animal model for investigating human nutrition and digestion. The similarity in microbial composition and diversity between the colon of pigs and the human intestines suggests that pigs serve as a viable model for understanding complex food interactions^11^. In particular, the colon of pigs closely mirrors that of humans, indicating its potential as a suitable model for gut. Seaweed phenolics have shown a positive effect when they reach the large intestine, where they can be broken down by the gut microbiome along with dietary fiber and other indigestible compounds. The gut microbiota also aids in the absorption of dietary minerals and produces short-chain fatty acids (SCFAs) and other bioactive metabolites^6,12^. An increase in the beneficial gut bacterial populations including *Bifidobacterium* and *Lactobacillus* enhances the beneficial effects on the microbiota and increases SCFA production^13^. For example, *Ecklonia radiata* stimulated the growth of beneficial microbes such as *Bifidobacterium* and *Lactobacillus* which are the most commonly recognized bacterial markers of prebiosis^14^. Another study reported an increase in *Bifidobacteria* and a higher ratio of *Firmicutes* to *Bacteroidetes*, and enhanced SCFA production following the addition of plant flavonoids such as rutin and quercetin in an in vitro model of human intestinal bacteria^15^. A recent study observed that the SCFAs including butyrate, propionate, and acetic acid, serve as an energy source for epithelial cells in the intestine, provide immunity against pathogens in the intestinal mucosa and inhibit the multiplication of colon cancer cells, and thereby help in improving gut health^6^. Thus, seaweeds can serve as a source of phenolics that are bioaccessible and bioavailable in the human gut. Moreover, research on the in vitro digestion and colonic fermentation of Australian beach-cast seaweeds is currently limited.

In this study, the brown beach-cast seaweeds examined were commonly and abundantly found on Australian south-east shores. Seaweeds are composed of approximately 80% water, making drying a key process in their concentration and recovery during bioprocessing. Phenolic compounds are heat sensitive to varying degrees depending on their structures, resulting in a loss of some of these valuable compounds during drying. The most effective drying method known for minimizing this degradation is freeze-drying^5^, and freeze-drying creates pores that can improve extraction yields^16^. The freeze-dried seaweed samples underwent successive in vitro digestion phases, including oral, gastric, and small intestinal digestion. Subsequently, the residue material was subjected to colonic fermentation at intervals of 0, 2, 4, 8, 16, 24 and 48 h. Post-digestion, the seaweed residue was quantified for phenolic compounds using high performance liquid chromatography (HPLC) combined with photodiode array detector (PDA). The phenolic compounds including phloroglucinol, gallic acid, pyrogallol, protocatechuic acid, 4-hydroxybenzoic acid, catechin and salicylic acid were quantified for their bioaccessibility. Additionally, gas chromatography with flame ionization detection (GC-FID) was used to detect short-chain fatty acids (SCFAs) namely propionic acid, acetic acid, butyric acid, iso-butyric acid, and valeric acid. This study has provided information on the in vitro digestion and colonic fermentation of phenolic content and antioxidant properties of the freeze-dried seaweeds and thus promotes and provides evidence toward further investigation of their application in food and pharmaceutical industries.

## Results and discussion

### Phenolic and antioxidant activity changes during in vitro digestion

#### Phenolic content

**Figure 1:**
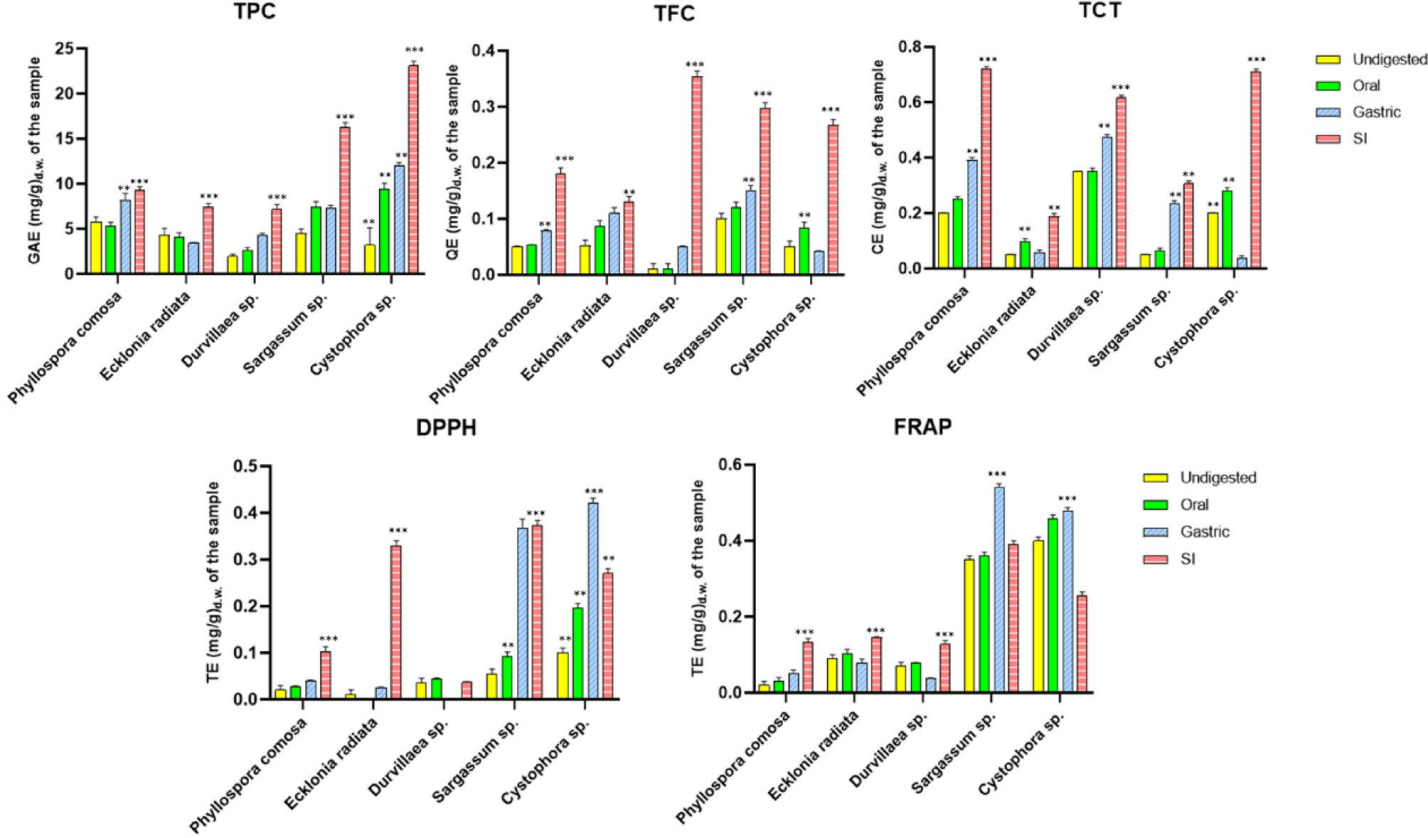
The evaluation of phenolic content and antioxidant potential of the freeze-dried seaweed sp., in vitro digests. The results were expressed as mean ± standard deviation (*n* = 3) on a dry weight basis which has been subtracted from the controls. In the graph, the yellow bar: undigested; the green bar: the oral phase; the blue bar: the gastric phase; the pink bar; the small intestinal phase. Abbreviations of GAE: gallic acid equivalents; QE: quercetin equivalents; CE: catechin equivalents; TE: Trolox equivalents; TPC: total phenolic content; TFC: total flavonoid content; TCT: total tannin content; DPPH: 2,2′-Diphenyl-1-picrylhydrazyl; FRAP: Ferric Reducing Antioxidant Power. **Statistically significant difference (*p* ≤ 0.05); ***Statistically very significant difference (*p* ≤ 0.01) within the species.

Figure 1 shows the total phenolic, flavonoid, and total tannin content of the five freeze-dried seaweed species. The total phenolic content (TPC) of all seaweed species showed a gradual increase during in vitro digestion, except in the gastric phase where a minor decrease in the phenolic content of *Ecklonia radiata* was detected. The total phenolic content released was between 1.95 to 5.76 mg GAE per g in the undigested phase, 2.65 to 9.34 mg GAE per g in the oral phase, 3.42 to 8.14 mg GAE per g in the gastric phase, and 7.15 to 23.1 mg GAE per g in intestinal phase. *Cystophora* sp., exhibited higher total phenolics released in the oral (9.34 mg GAE/g), gastric (12.04 mg GAE/g) and intestinal (23.1 mg GAE/g) phases. The observed values were higher in the TPC assay than those measured in other in vitro assays, likely because the Folin-Ciocalteu method detects certain peptides and proteins, thereby increasing the observed TPC values^17^.

The release of total flavonoid content (TFC) and the total tannin content (TCT) gradually increased during the stimulated digestion but significantly increased (*p* < 0.05) in the intestinal phase. For TFC, the flavonoid release in the undigested phase ranged from 0.05 to 0.1 mg QE per g, the oral phase was 0.053 to 0.12 mg QE per g, the gastric phase was 0.042 to 0.149 mg QE per g and intestinal phase were 0.13 to 0.35 mg QE per g. *Durvillaea* sp., had a high release of flavonoids throughout the in vitro digestion process. Similarly, in the TCT assay, the tannin content ranged from 0.05 to 0.35 mg CE per g in the undigested phase, 0.063 to 0.352 mg CE per g in the oral phase, 0.036 to 0.474 mg CE per g in the gastric phase and 0.188 to 0.72 mg CE per g in the intestinal phase. In TCT, *P. comosa* gradually increased in the oral, gastric, and intestinal phases. However, the *Durvillaea* sp., had a high release of TCT content which decreased in the intestinal phase. Similarly, in the gastric phase, both *Cystophora* sp. and *Ecklonia radiata* showed lower values compared to other species in the TFC and TCT assays.

In the oral phase, the simple phenolics are released due to the short time. These simple phenolics include compunds like phlorotannin, gallic acid, protocatechuic acid and 4-hydroxybenzoic acid. As the food matrix moves to the gastric phase, HCl and pepsin lead to degradation, oxidation, or polymerization of the phenolic compounds^18^. However, Chandrasekara and Shahidi^19^ reported that in the gastric phase, protein-bound phenolics are released as proteins undergo digestion. Additionally, the study conducted by Feng et al.^20^ reported that chlorogenic acid undergoes hydrolysis in the gastric environment, and the hydrolysate of chlorogenic acid remains detectable even after gastric digestion.

In the intestinal phase, our study observed a high release of phenolic compounds, flavonoid, and tannin content. The reason might be that alkaline pH in the intestinal environment facilitates the solubility and stability of the phenolic compounds, especially those that are less stable in an acidic environment. Attri et al.^22^ reported that pancreatin enzyme affected the binding of the phenolic compounds with the food matrix, resulting in the release of phenolics in the intestinal phase. Our study demonstrates that seaweeds release different amounts of phenolics in each phase. The difference might be attributed to chemical compositions, dietary fibre, hydrophobic interactions, hydrogen bonding, and covalent bonds^23^. In conclusion, *Cystophora* sp., had high phenolic content released in oral, gastric and intestinal phase when compared to other seaweeds in this study. Regarding TFC, *Durvillaea* sp. exhibited high flavonoid release in the oral, gastric and intestinal phases, while *P. comosa* showed high TCT release in these same phases.

#### Antioxidant activity

Our previous studies have shown that phenolics compounds extracted from freeze-dried seaweed species exhibit strong antioxidant potential^4,5^. However, there is insufficient information available regarding the release of phenolic compounds during in vitro digestion of seaweeds that possess antioxidant properties. To investigate the antioxidant potential, various methodologies are available. In this study, we assessed the antioxidant activity via DPPH and FRAP assays in the oral, gastric, and intestinal phases, which are depicted in Fig. 1**.**

The DPPH radical scavenging method is commonly used to evaluate antioxidant potential due to its stability and accessibility of its reagent, making it more reliable than other chromogenic reagents^24^. In this study, there was a continuous significant increase in the antioxidant potential of every phase in *P. comosa*, and *Sargassum* sp. However, the antioxidant potential was not detected in the oral phase of *E. radiata.,* and the gastric phase of *Durvillaea* sp., whereas, in *Cystophora* sp., the intestinal phase was significantly lower than the gastric phase. The variation in the antioxidant potential might be attributed to the chemical characteristics and specific structural features of the individual phenolics present^19^.

The FRAP assay is a simple, fast, and cost-effective method and does not require specialised equipment^24^. In this assay, *P. comosa*, displayed a gradual increase in antioxidant potential from the oral to the intestinal phase. In *E. radiata* and *Durvillaea* sp., the antioxidant potential was higher in the gastric phase. In *Sargassum* sp., and *Cystophora* sp., the gastric phase displayed a higher release of phenolic compounds than the intestinal phase. The higher antioxidant potential observed during the oral and gastric phases may be because they exist in free and soluble form^25^. Similarly, dried raspberry fruit exhibited the same trend, with a higher release of antioxidants in the gastric phase than in other phases^25^. Some uncertainty in the results may arise from potential molecular changes in phenolics or their interaction with other constituents in the digestion samples during the antioxidant assay^17^. In addition, phenolic compounds that are not extracted or absorbed in the small intestine can undergo further metabolism by the gut microbiota in the large intestine. In conclusion, in the DPPH assay, *Cystophora* sp., exhibited high antioxidant potential in the gastric phase. Similarly, *Sargassum* sp., and *Cystophora* sp., demonstrated high FRAP values in the gastric phase.

### Phenolic and antioxidant activity changes during colonic fermentation

#### Phenolic content

**Figure 2:**
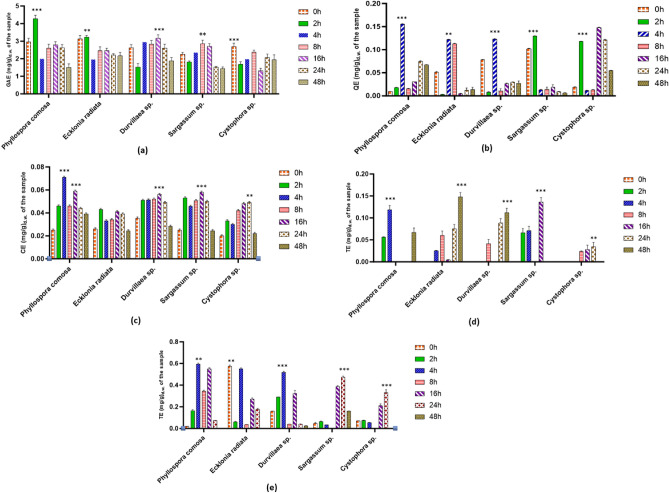
The phenolic content and antioxidant potential estimation of freeze-dried seaweed sp., after complete colonic fermentation. (**a**) TPC; (**b**) TFC; (**c**) TCT; (**d**) DPPH; (**e**) FRAP. The results were expressed as mean ± standard deviation (*n* = 3) on a dry weight basis which has been subtracted from the controls. Orange bar: 0 h; green bar: 2 h; blue bar: 4 h; pink bar: 8 h; purple bar: 16 h; red bar: 24 h; brown bar: 48 h. GAE: gallic acid equivalents; QE: quercetin equivalents; CE: catechin equivalents; TE: Trolox equivalents. **Statistically significant difference (*p* ≤ 0.05) within the species; ***Statistically very significant difference (*p* ≤ 0.01) within the species.

The effects of in vitro colonic fermentation on bioactive compounds and the antioxidant potential of the digested seaweed samples were investigated, as illustrated in Fig. 2. During colonic fermentation, the total phenolic content in *P. comosa* and *E. radiata* increased gradually at 2 h and then decreased progressively. In the *Durvillaea* sp., and *Sargassum* sp., a significant release of phenolic compounds was observed at 16 h. Meanwhile, in the TFC assay, the maximum flavonoid was seen at 4 h in *P. comosa*, *E. radiata*, and *Durvillaea* sp. *Cystophora* sp., released higher flavonoid content at 16 h (0.148 mg QE per g), followed by a decline at 24 and 48 h. In the total tannin content, the maximum tannin was extracted in *P. comosa* at 4 h intervals (0.07 mg CE per g). All five freeze-dried seaweed species had a similar trend where tannin content decreased from 16 to 48 h. Ma et al.^26^ reported that between 0.5 and 8 h of fermentation time, there was an overall increase in phenolic compounds as the bonds between the phenolics and the cellular wall are broken down due to microbial fermentation and released in their free form. Attri et al.^27^ reported that after 36 h the phenolic compounds rapidly decreased due to the degradation of the food matrix, which was consistent with our data. In conclusion, *P. comosa*, had high phenolic content released at 2 h, and high total flavonoid and tannin content released at 4 h.

#### Antioxidant activity

In this study, the antioxidant potential was observed and estimated in every species using the DPPH and FRAP assays, with results shown in Fig. 2. The antioxidant mechanisms in the DPPH assay involves providing hydrogen atoms to neutralize the DPPH radical, while in the FRAP assay, it involves single electron donation. Antioxidants, which are free radical scavengers, protect cells and tissues from damage and prevent chronic diseases^28^. However, antioxidant potential was not consistently observed across all time intervals. The antioxidant potential measured using the DPPH assay was high at 48 h in *E. radiata* and *Durvillaea* sp., whereas in *P. comosa* and *Sargassum* sp., the antioxidant potential was high at 4 and 16 h. For *P comosa*, *E radiata*, and *Durvillaea* sp., in FRAP assay the antioxidant potential was high at 4 h. Time-dependent variations in antioxidant activity might result from structural changes in phenolic compounds, which could lead to the formation of new compounds that may have higher or lower antioxidant capacities^29^. The antioxidant potential of *Sargassum* sp., and *Cystophora* sp., was notably high at 24 h.

### Bio-accessibility of selected phenolic compounds in seaweeds.

Bioaccessibility refers to the proportion of phenolics that are absorbed by the epithelial layer of the gastrointestinal tract^30^. In this study, we observed the amount of phenolic compounds available in the oral, gastric, small intestine phases, and during the colonic fermentation using HPLC–PDA. In our study, the bioaccessibility of phenolic compounds including phlorotannins, gallic acid, pyrogallol, protocatechuic acid, 4-hydroxybenzoic acid, catechin, and salicylic acid that are available to be absorbed was determined with results shown in Table 1. Phloroglucinol was detected in the gastric phase of *P. comosa*, *E. radiata*, and *Sargassum* sp., indicating its bioaccessibility. Similarly in the intestinal phase, the bioaccessibility of phloroglucinol was higher and observed in *P. comosa*, *Sargassum* sp., and *Cystophora* sp., but undetectable in *E. radiata*. However, the bioaccessibility of this compound in the colonic phase was lower compared to the intestinal phase. In contrast, Corona et al.^31^ found that phlorotannin had low absorption in the small intestine due to the production of conjugate metabolites such as glucuronides and sulphates. Phloroglucinol is formed from catechin through the conversion of flavonoid monomers to a quinonic form, followed by in vitro polymerisation*,* which results in decreased solubility^32^.

**Table 1:** Estimation of bio accessibility after complete in vitro digestion and colonic fermentation for phenolic compounds in freeze-dried seaweed sp.

Compounds	Oral BIA (%)					Gastric BIA (%)					SI BIA (%)					Colonic BIA (%)				
	PC^**a**^	ER	DS	SS	CS	PC	ER	DS	SS	CS	PC	ER	DS	SS	CS	PC	ER	DS	SS	CS
Phloroglucinol	36.90	–	–	44.01	–	76.65	68.79	–	26.16	–	139.72	–	–	346.25	171.88	65.62	–	50.73	60.89	–
Gallic acid	37.92	–	–	–	43.66	–	81.67	0.00	111.01	61.23	0.00	134.95	–	136.97	277.51	40.29	41.18	–	39.46	–
Pyrogallol	–	–	–	–	–	–	–	0.00	154.47	0.00	–	0.00	–	0.00	–	0.00	0.00	0.00	0.00	–
Protocatechuic acid	35.43	47.59	42.93	20.25	39.63	–	–	–	37.09	32.80	129.89	–	63.56	69.23	–	38.09	60.85	26.31	23.00	26.83
4-hydroxybenzoic acid	–	41.48	41.3	42.53	42.6	–	–	–	–	82.38	–	153.70	151.7	158.70	–	–	46.25	46.51	46.40	48.90
Catechin	0.00	–	0.00	0.00	7.96	0.00	–	–	–	–	–	–	0.00	–	0.00	–	–	–	0.00	–
Salicylic acid	–	–	–	0.00	–	0.00	–	–	12.44	0.00	–	0.00	0.00	–	0.00	0.00	1.64	–	0.00	0.00
Total phenolic compounds	13.69	35.55	260.84	60.27	52.56	5.48	37.01	613.80	99.02	80.49	17.88	170.90	863.26	325.18	60.48	16.48	55.51	296.70	89.78	35.18

^a^Seaweed samples mentioned in abbreviations are *Phyllospora comosa* “PC”; *Ecklonia radiata* “ER”, *Durvillaea* sp.,”DS”; *Sargassum* sp., ”SS” and *Cystophora* sp.,”CS”. Abbreviations of bioaccessibility “BIA”. ‘–’ shows that compound was not detected during analysis while “0.00” means the bioaccessibility of the compound is too low to quantify using the equation.

Gallic acid showed high bioaccessibility in the gastric phase, which continued into the intestinal phase. Gallic acid is derived from protocatechuic acid. In in vitro digestion, enzymes such as esterases and glycosidases cleave any ester or sugar groups that may be attached to the gallic acid molecule^33^. During the colonic fermentation phase, the bioaccessibility of the gallic acid was approximately 40% for *P. comosa*, *Durvillaea* sp., and *Sargassum* sp. In the large intestine, the metabolic activity of the microbiota leads to changes in the phenolic compounds due to the formation of new metabolites^34^.

**Figure 3:**
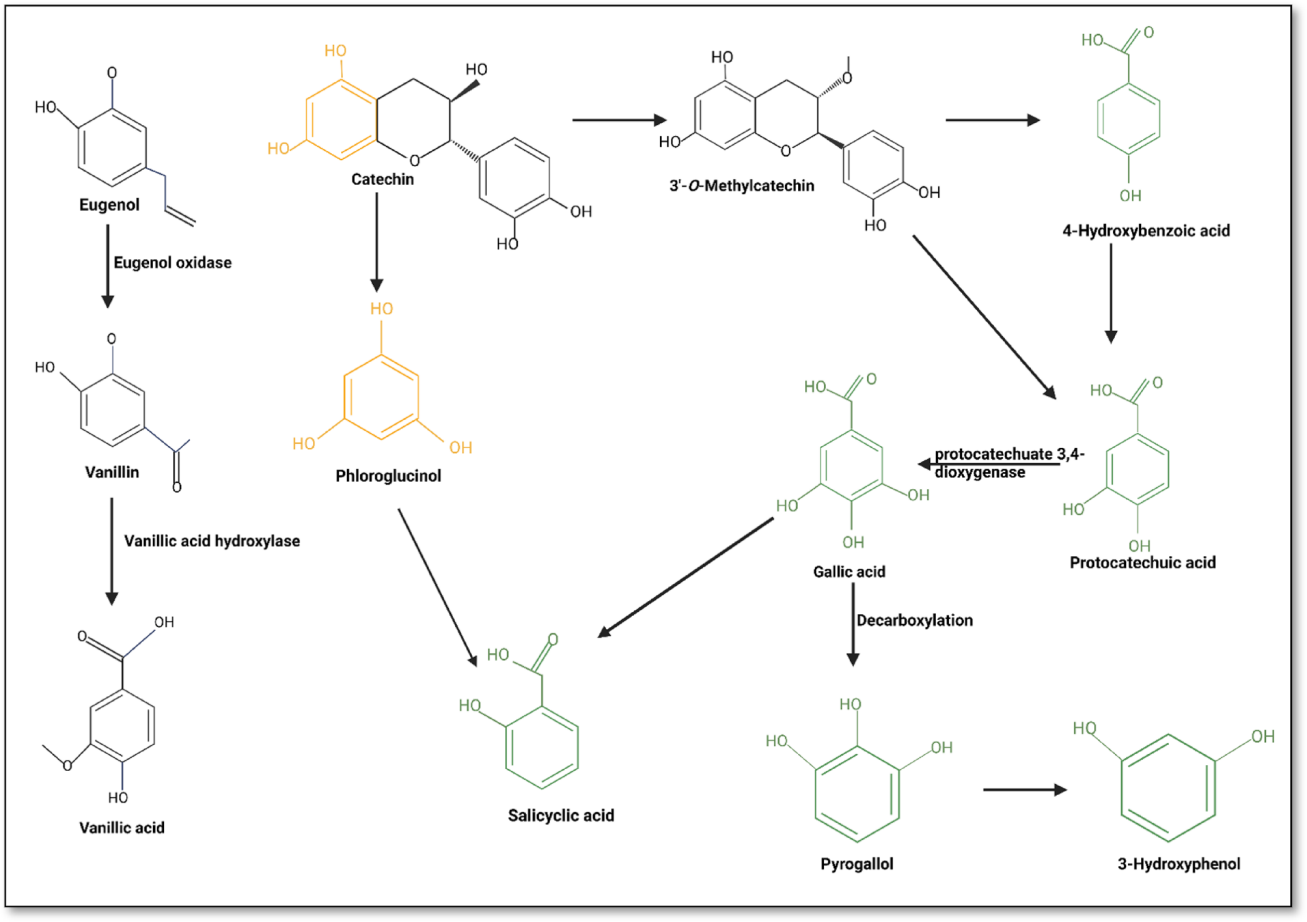
Possible metabolic pathway of dietary phenolic compounds^,^^36,37–39^.

The conversion of 4-hydroxybenzoic acid to protocatechuic acid, catalyzed by 4-hydroxybenzoate 3-monooxygenase (Fig. 3), was observed in the oral phase across all seaweed species, with bioaccessibility ranging from 35 to 48%. In our study, *P. comosa* exhibited the highest bioaccessibility, at 129.89%, in the small intestine, but this was significantly reduced to 38% during colonic fermentation. The metabolic transformation of polyhydroxylated phenolic compounds, including flavonoids, contributed to the production of 4-hydroxybenzoic acid^35^. In the oral phase, the compound was detected in all species except *P. comosa*, where the bioaccessibility level was approximately 40%. Interestingly, the highest bioaccessibility was exclusively observed in the small intestinal phase for *E. radiata*, *Durvillaea* sp., and *Sargassum* sp., suggesting distinct metabolism in the small intestine. However, bioaccessibility was reduced during the colonic fermentation phase. As previously mentioned, both the intestinal phase and colonic fermentation have the ability to metabolize new phenolic compounds^34^.

### Recovery and residual index

In our study, we calculated the soluble and insoluble fraction of selected phenolic compound in the seaweed species, as shown in Table 2. We observed a significant difference (*p* < 0.05) in phenolic compound recovery between the intestinal and colonic phases. The recovery index serves as a measure of the presence of phenolic compounds in the digesta of the oral, gastric and intestinal phases^40^. Notably, the seaweed species *P. comosa*, *Sargassum* sp., and *Cystophora* sp., exhibited a 100% recovery of phloroglucinol compounds following intestinal digestion. This result was consistent with the 100% recovery observed for gallic acid, protocatechuic acid and 4-hydroxybenzoic acid in specific seaweed species as shown in Table 2. However, in the case of *P. comosa*, protocatechuic acid and 4-hydroxybenzoic acid showed reduced recovery rates of 30% and 40%, respectively, in the colonic phase. Pyrogallol and catechin were not detected in the recovery index of both intestinal and colonic phases. On the other hand, salicylic acid was detected in the colonic recovery index.

**Table 2:** Estimation of recovery (%) and residual digesta index (%) after complete in vitro digestion and colonic fermentation for phenolic compounds of beach-cast brown seaweed spp.

Compounds	Intestinal recovery index (%)					Colonic total recovery (%)					Residual intestinal digesta index (RID%)					Residual colonic digesta index (RCD%)				
	PC	ER	DS	SS	CS	PC	ER	DS	SS	CS	PC	ER	DS	SS	CS	PC	ER	DS	SS	CS
Phloroglucinol	143.78	4.35	4.13	350.33	175.98	73.76	9.80	58.86	68.62	7.73	4.06	4.35	4.13	4.08	4.1	8.14	9.8	8.13	7.73	7.73
Gallic acid	–	138.67	–	136.90	277.51	44.48	51.13	4.21	43.68	4.18	–	3.71	–	–	–	4.18	9.95	4.21	4.21	4.18
Pyrogallol	–	–	–	–	–	–	–	–	–	–	–	–	–	–	–	0.00	0.00	0.00	0.00	0.00
Protocatechuic acid	129.89	–	63.56	69.23	–	38.08	60.84	26.31	23.06	–	–	–	–	–	–	–	–	–	–	–
4-hydroxybenzoic acid	–	153.70	151.73	158.70	–	–	46.25	46.51	46.42	48.98	–	–	–	–	–	–	–	–	–	–
Catechin	–	–	–	–	–	–	–	–	–	–	–	–	–	–	–	0.00	0.00	0.00	0.00	0.00
Salicylic acid	–	–	–	–	–	–	1.64	–	–	–	–	–	–	–	–	–	–	–	–	–
Total Phenolic Compounds	180.40	321.46	176.60	557.50	411.5	103.38	168.10	613.80	296.60	55.54	2.68	8.84	11.05	6.65	3.73	8.14	21.6	84.6	19.46	10.84

Seaweed samples mentioned in abbreviations are *Phyllospora comosa* “PC”; *Ecklonia radiata* “ER”, *Durvillaea* sp.,”DS”; *Sargassum* sp.,”SS” and *Cystophora* sp.,”CS”. ‘–’ shows that compound was not detected during the analysis while “0.00” means the bioaccessibility of the compound is too low to quantify using the equation.

The residual recovery of the phenolic compounds was zero or not detected in the intestinal phase, likely due to the presence of enzymes that metabolise these compounds. The compounds found in the residual form in both the intestinal and colonic phases are phloroglucinol and gallic acid. Pyrogallol and catechin had zero indexes as their concentration was too low to be quantified using the equation. If the residual in the colonic phase remains zero, it could indicate that more time is needed for the extraction of phenolic compounds.

### Short chain fatty acids

Short-chain fatty acids (SCFAs) are a group of organic acids, also known as volatile fatty acids, that have a carbon chain length of 1–6. The most common SCFAs are propionic acid, acetic acid, and butyric acid^41^. SCFAs are formed through the process of anaerobic fermentation of dietary fibers and resistant starches that reach the colon undigested^12^. SCFAs benefit human health by providing energy for colon cells, reducing inflammation in the colon region, modulating the release of insulin levels and reducing appetite to decrease the intake of the calorie^42^. Research by He et al.^41^ indicates that diet and the gut microbiome are the main factors that affect the production of short-chain fatty acids. Thus, maintaining a balanced gut microbiota is essential to support the production of SCFAs. Additionally, biotransformed polyphenols have the potential to modulate the gut microbiota and contribute to beneficial health benefits.

**Figure 4:**
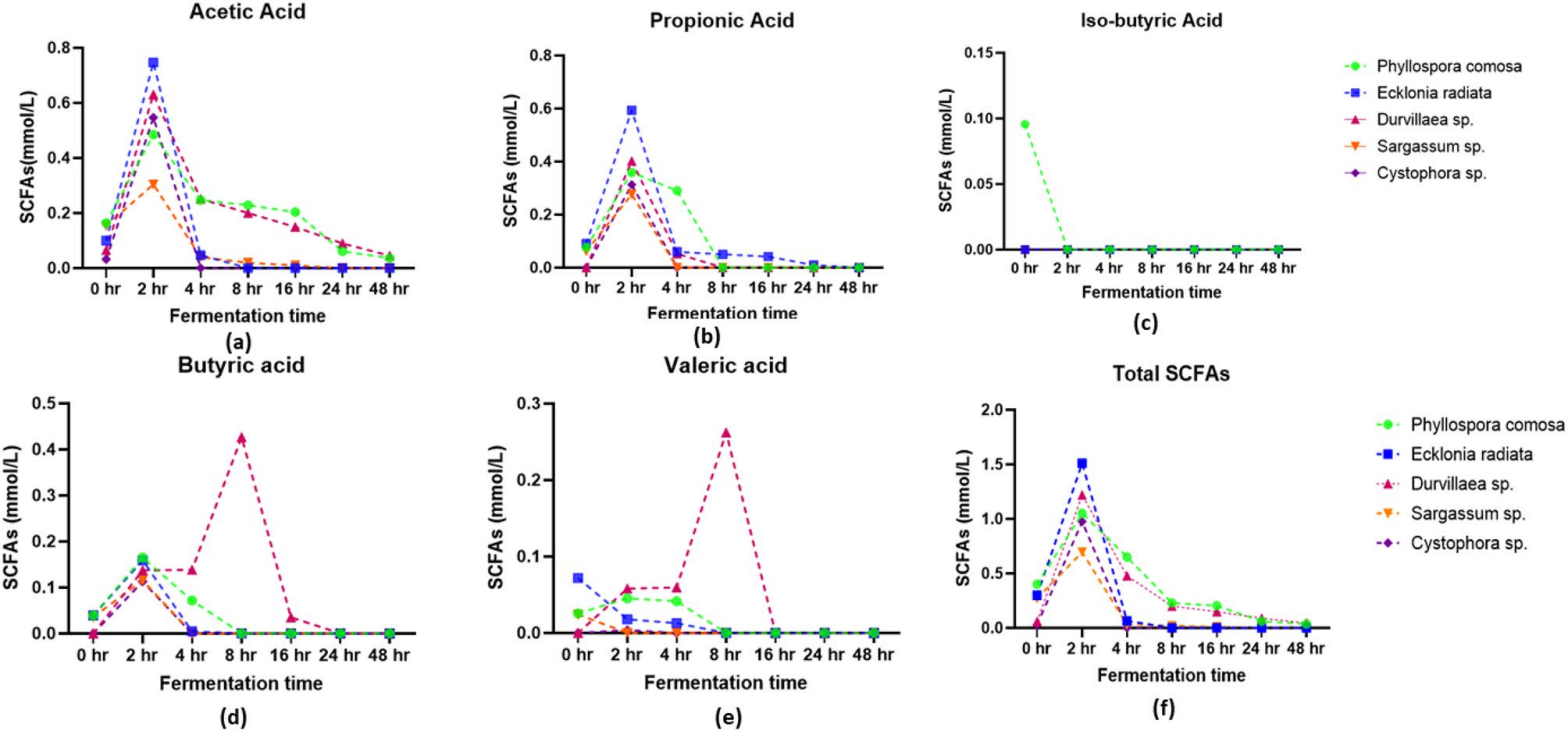
The SCFAs production in different seaweed digesta. (**a**) Acetic acid levels in freeze-dried seaweeds digesta; (**b**) propionic acid level in seaweed digesta; (**c**) iso-butyric acid level in seaweed digesta; (**d**) butyric acid level in seaweed digesta; (**e**) valeric acid level in seaweed digesta; (**f**) total short-chain fatty acid level in seaweed digesta.

In our study, we observed the production of SCFAs including propionic acid, acetic acid, butyric acid, iso-butyric acid, and valeric acid in five seaweed species digesta (Fig. 4). We observed that the production of acetic acid and propionic acid were relatively high at 2 h with values ranged between 0.3 to 0.74 mmol L^−1^. *E. radiata* exhibited the highest levels of acetic and propionic acid followed by *Durvillaea* sp. Acetate is formed via the Wood–Ljungdahl pathway and butyrate is formed from two molecules of acetate^12^. The production of acetate and propionate is associated with *Bacteroides* spp. Interestingly, we observed the production of iso-butyric acid only in *P. comosa*, at the beginning of the colonic fermentation (0 h), likely due to the sudden increase in certain microbial populations capable of producing isobutyric acid^43^. On the other hand, butyric acid production was high in all the seaweed digesta at 2 h; however, *Durvillaea* sp., digesta showed high production at 8 h and rapidly declined at 16 h. In the case of valeric acid, production gradually increased in *Durvillaea* sp., and was high at 8 h, and gradually decreased afterward. The results of Wang et al.^23^ is inconsistent with our results, as in their study the production of SCFAs increased after 16 h, which may be due to slower fermentation of the dietary fibre by the gut microbiota. *Bifidobacterium* and *Firmicutes phylum* bacteria produce butyric acid by first fermenting carbohydrates and then producing acetic acid^44^. Additionally, Louis and Flint^45^ reported that butyrate supplies energy to the gut mucosa and also inhibits histone deacetylases. The variations in SCFAs production observed in our study may be due to factors such as particle size, water solubility, molecular weight, and bacterial digestibility of the dietary fibers^46^. These factors can influence the fermentation process and the resulting SCFA profile in the seaweed digesta.

## Conclusion

The release of phenolic compounds from seaweed digesta occurs continuously throughout in vitro digestion and colonic fermentation. In *Cystophora* sp., enzymes facilitated the release of high amount of phenolic compounds in the oral, gastric, and the small intestinal phase. In contrast, *Durvillaea* sp., exhibited high flavonoid content in the small intestinal phase and high tannin content in the gastric phase. The antioxidant potential of *Sargassum* sp., and *Cystophora* sp., was significantly higher in the gastric and intestinal phases compared to other species. The bioacessibility of the compounds protocatechuic acid was detected in all seaweed species in oral phase. In gastric phase, *Sargassum* sp., exhibited the highest bioacessibility of gallic acid compared to other species. However, in the small intestine, *Sargassum* sp., showed high bioaccessibility of phlorotannin. As the food matrix moved to the large intestine, the microbiota degraded and dietary fibre fermented, improving the bioaccessibility of the specific phenolic compounds including protocatechuic acid, and 4-hydroxybenzoic acid. SCFAs production was detected in all the seaweed species, with acetic acid being the major SCFA detected in all species investigated. Maximal acetic and propionic acid were detected at 2 h for all seaweed species. However, *Durvillaea* sp., exhibited highest butyric and valeric acid at 8 h. According to our study, *Durvillaea* sp., *Sargassum* sp., and *Cystophora* sp., are good sources of phenolic compounds when indigested. However, it would be beneficial to further investigate the effects of different drying methodologies on in vitro digestion and colonic fermentation processes. Additionally, further research is required to understand the impact and mechanisms of microbiota on phenolic biotransformation, and vice versa.

## Materials and methods

### Sample collection and sample preparation

*Phyllospora comosa*, *Ecklonia radiata*, *Durvillaea* sp., *Sargassum* sp., and *Cystophora* sp., seaweeds were collected from Queenscliff Harbour (38°15′54.0″S 144°40′10.3″E), Victoria, Australia and collected on 17th February 2022 (12:55 h) at the temperature of 24 °C (summer). The respective seaweeds were randomly collected from seashore, irrespective of their age, shape and size, and were identified at Deakin Marine Institute, Queenscliff, Victoria, Australia. Fresh seaweed samples were first washed with tap water, followed by Milli-Q water, to eliminate any extraneous impurities. The seaweed samples were manually cut into smaller pieces, approximately 1–3 cm each, using a stainless-steel food-grade knife. The fresh seaweed samples were freeze-dried. The seaweed samples were frozen at − 70 °C for 24 h in a Thermo scientific freezer. The frozen samples were placed in the freezer dryer at − 60 °C for 72 h, following the procedure described by Badmus et al.^47^. The sample was ground into a fine powder using a grinder (Cuisinart Nut and Spice grinder 46,302, Melbourne, VIC). The dried samples were stored in the cold room (4 °C).

### Chemicals and reagents

Analytical grade standards and chemicals for this study were procured from Sigma-Aldrich Chemicals (Castle Hill, NSW, Australia). Standards employed in this study, including catechin, gallic acid, phloroglucinol, 4-hydroxybenzoic acid, salicylic acid, and protocatechuic acid were utilized for HPLC–PDA analysis to determine bioaccessibility. The chemicals utilized in the in vitro digestion included _L_-cysteine, calcium chloride, ammonium carbonate, potassium chloride, yeast extract, sodium bicarbonate, sodium chloride, magnesium sulphate heptahydrate, potassium dihydrogen phosphate, sodium hydroxide, hydrochloric acid, pepsin, soluble starch, peptone, tryptone, potassium chloride, pectin, mucin, casein, tween®-80 and bile salts. Chemicals used in the in vitro phenolic and antioxidant assays included sodium carbonate anhydrous, Folin & Ciocalteu’s phenol reagent, hexahydrate aluminium chloride, sodium acetate, vanillin, 2,2′-diphenyl-1-picrylhydrazyl (DPPH), Iron (III) chloride (FeCl_3_) and 2,4,6-tripyridyl-s-triazine (TPTZ). The solvents used were methanol, ethanol, and sulphuric acid. The pancreatin and α-amylase enzymes used for in vitro digestion were purchased from the Assay Matrix Pty Ltd., Australia.

### Extraction of phenolic compounds from freeze-dried seaweed.

#### Free and bound phenolic extraction

Free phenolics were extracted by homogenising 1 g of freeze-dried sample in 10 mL of water for 30 s at 10,000 rpm using an Ultra-Turrax T25 Homogenizer (IKA, Staufen, Germany)^48^. Subsequently, the homogenised samples were incubated in the shaking incubator (ZWYR-240 incubator shaker, Labwit, Ashwood, VIC, Australia) for 12 h at 4 °C/120 rpm. After extraction, the samples were subjected to centrifugation at 8000 rpm for 15 min under 4 °C using Hettich Refrigerated Centrifuge (ROTINA380R, Tuttlingen, BadenWürttemberg, Germany). The resulting supernatant fluid was then filtered through a 0.45 µm syringe filter (Thermo Fisher Scientific Inc., Waltham, MA, USA) to obtain the free phenolic extracts. The sample residues were air-dried for 72 h. The residues were washed 3 times with their respective solvents and then further analysed for bound phenolics.

One gram of residue was mixed with 5 mL of 2 N NaOH in a screw-capped test tube. The sample was neutralized (pH 7) with 2 N HCl and added with 5 mL of water^49^. The samples were placed in a shaking incubator and incubated for 16 h at 120 rpm at 4 °C (ZWYR-240 incubator shaker, Labwit, Ashwood, VIC, Australia). After incubation, the samples were centrifuged at 8000 rpm for 15 min at 4 °C using a Hettich Refrigerated Centrifuge (ROTINA380R, Tuttlingen, BadenWürttemberg, Germany). A 0.45 µm syringe filter was used to filter the supernatant. The filtered supernatant was collected as bound phenolic extracts and stored at − 20 °C for further analyses.

### In vitro gastrointestinal digestion and colonic fermentation

Following the protocol by Gu et al.^50^, the dried seaweed samples underwent in vitro digestion using the harmonized INFOGEST 2.0 protocol. For the oral phase, 2.5 mL of the sample (containing 0.5 g of dried seaweed) and 2.5 mL of water were combined with 75 U/mL salivary α-amylase and stimulated oral fluid (SOF) in 1:1 ratio (*v/v*), followed by incubation for 2 min with constant shaking at 37 °C. Moving to the gastric phase, 2.5 mL of the sample extracted from the oral phase was mixed with stimulated gastric fluid (SGF) (1:1, *v/v*) and 2000 U/mL porcine pepsin. In this phase, the mixture’s pH was adjusted to 3.0 by the addition of HCl and incubated at 37 °C for 2 h. Finally, in the last phase of digestion, 2.5 mL of the mixture was taken from gastric digestion and was neutralised to pH 7.0. This was achieved by adding 100 U/mg trypsin, stimulate intestinal fluid (SIF) (1:1, *v/v*) and 10 mM bile salt. The small intestinal phase was incubated at 37 °C for 2 h. After each in vitro digestion phase, the sample was frozen at − 80 °C to suspend the enzymatic reactions.

Following the protocol by Gu et al.^50^, in vitro colonic fermentation involved the use of pig feces as an alternative to human feces. Ten mixed male and female large landrace grower white pigs, each weighing 50 kg and raised at Diamond Valley Pork's animal house in Laverton North, VIC, Australia, were fed a standard diet for two weeks. The collected feces samples were freshly pooled and thoroughly mixed within an anaerobic chamber. For the preparation of faecal media, 20 g of faeces were mixed with 80 g of 0.1 M sterilized phosphate buffer (pH 7.0). The resulting faecal media was then filtered and ready to use. The sample (0.2 g), basal media (2 mL) and faecal media (2 mL) were prepared (1:10:10, *w/v/v*) in seven sets of tubes flushed with nitrogen. These seven sets of tubes were incubated for 0, 2, 4, 8, 16, 24, and 48 h, respectively. After the incubation period, the mixture was centrifuged for 10 min at 5 °C at 10,000 g. The collected supernatant was then used for the analysis of phenolic and short-chain fatty acids production.

### Estimation of phenolic content and antioxidant capacity

#### Determination of total phenolic content (TPC)

The total phenolic content was estimated using Folin-Ciocalteu’s method, following the protocol described by Mussatto et al.^51^, where 25 µL of extract, 25 µL Folin-Ciocalteu’s reagent solution, and 200 µL water were mixed in a 96-well plate (Costar, Corning, NY, USA). The plate was incubated in the dark room at room temperature (~ 25 °C) for 5 min, and then 25 µL of 10% (*w:w*) sodium carbonate was added and incubated at 25 °C for 60 min. Absorbance was measured at 765 nm using a 96-well plate reader (Thermo Fisher Scientific, Waltham, MA, USA). Concentration ranging from 0 to 200 µg/mL of gallic acid was prepared as a standard curve and the TPC content was expressed in mg GAE/g of the sample of dry weight_(d.w.)_.

#### Determination of total flavonoid compounds (TFC)

Total flavonoid content (TFC) was measured using the modified aluminum chloride method, as described in Ali et al.^52^. 80 µL extract followed by 80 µL of 2% aluminium chloride and 120 µl of 50 g/L sodium acetate solution were added to the 96-well plate. The plate was then incubated in the dark for 2.5 h. Absorbance was measured at 440 nm using a 96-well plate reader. The concentration ranging from 0 to 50 µg/mL for the quercetin calibration curve was used to determine TFC and expressed in mg QE/g _d.w_.

#### Determination of total tannin content (TCT)

Total tannin content was determined using the vanillin sulphuric acid method, slightly modified from the method described by Ali et al.^52^. 25 µL of sample extract was added to a 96-well plate, followed by the addition of 25 µL of 32% sulphuric acid and 150 µL of 4% vanillin solution. The mixture was incubated for 15 min, and the absorbance was measured at 500 nm using a 96-well plate reader. The TCT was expressed in mg CE/g _d.w_.

#### 2,2′-Diphenyl-1-picrylhydrazyl (DPPH) assay

Free radical scavenging activity of the seaweed samples was estimated using the DPPH method, slightly modified from the protocol described by Nebesny and Budryn^53^. Firstly, 4 mg of DPPH was dissolved in 100 mL of analytical-grade methanol to prepare the DPPH radical solution. Then, 40 µL of sample extract was mixed with 260 µL of DPPH solution in a 96-well plate and incubated at 25 °C for 30 min. The absorbance was measured at 517 nm using a 96-well plate reader and the radical scavenging activity was expressed as mg TE/g _d.w_.

#### Ferric reducing antioxidant power (FRAP) assay

This assay has been used to estimate the antioxidant capacity in marine seaweeds with some modifications as described by Benzie and Strain^54^. To prepare the FRAP dye, a mixture of 20 mM Fe [III] solution, 10 mM 2,3,5-Triphenyltetrazolium chloride solution, and 300 mM sodium acetate solution was prepared in a ratio of 1:1:10. Next, 20 µL of the extract and 280 µL prepared dye were added to a 96-well plate and the mixture was incubated at 37 °C for 10 min. The absorbance was measured at 593 nm using a 96-well plate reader, and the antioxidant potential was expressed as mg TE/g _d.w_.

### Quantification of phenolic compounds

Quantification of seaweed phenolics was performed using an Agilent 1200 series high-performance liquid chromatography (HPLC) system (Agilent Technologies, CA, USA), equipped with a photodiode array (PDA) detector^50,55^. A reversed-phase column of particle size 4 µm Synergi Hydro-RP (250 × 4.6 mm i.d.) protected with Phenomenex 4.0 × 2.0 mm i.d., C18 ODS guard column was used in our study. The injection volume was 25 μL. Mobile phases A and B consisted of water/acetic acid (98:2, *v/v*) and acetonitrile/water/acetic acid (50:50:2, *v/v/v*), respectively. The flow rate was 0.8 mL/min, and the gradient profile varied as follows: 90%–10% B (0–20 min), 75%–30% B (20–30 min), 65%–35% B (30–40 min), 45%–55% B (40–60 min), 90%–10% B (60–61 min), 90%–10% B (61–66 min). The photodiode array (PDA) detector was set to simultaneously detect wavelengths of 280, 320, and 370 nm. Data analysis was carried out using Empower Software (2010).

### Indices of in vitro digestion and colonic fermentation

#### Bioaccessibility of selected phenolics compounds

Each selected seaweed phenolics’ bioaccessibility can be estimated by calculating the proportion of the quantity of the phenolics released in each digestion phase and the quantity of phenolics released in the undigested phase^30^.

$${\text{Oral}}\;{\text{Bioaccessibility }}\left( \% \right)\, = \,\left( {{\text{Oral}}\;{\text{fraction}}\;{\text{of}}\;{\text{phenolics/Total}}\;{\text{phenolics}}} \right)\, \times \,{1}00.$$

$${\text{Gastric}}\;{\text{Bioaccessibility }}\left( \% \right)\, = \,\left( {{\text{Gastric}}\;{\text{fraction}}\;{\text{of}}\;{\text{phenolics/Total}}\;{\text{phenolic}}\;{\text{content}}} \right)\, \times \,{1}00.$$

$${\text{Intestinal}}\;{\text{ Bioaccessibility }}\left( \% \right) \, = \, \left( {{\text{Intestinal }}\;{\text{fraction}}\;{\text{ of}}\;{\text{ phenolics}}/{\text{Total}}\;{\text{ phenolic }}\;{\text{content}}} \right) \, \times { 1}00.$$

$${\text{Colonic}}\;{\text{Bioaccessibility }}\left( \% \right)\, = \,\left( {{\text{Colonic }}\;{\text{fraction }}\;{\text{of }}\;{\text{phenolics/Total }}\;{\text{phenolic }}\;{\text{content}}} \right)\, \times \,{1}00.$$

### Estimation of recovery index

The recovery index was calculated using a formula that utilized the fractions of soluble and insoluble phenolics^30^.

$${\text{Soluble }}\left( \% \right)\, = \,\left( {{\text{Content }}\;{\text{in}}\;{\text{ soluble }}\;{\text{fraction/Total}}\;{\text{ phenolic}}\;{\text{ content}}} \right)\, \times \,{1}00.$$

$${\text{Insoluble }}\left( \% \right)\, = \,\left( {{\text{Content }}\;{\text{in}}\;{\text{ insoluble}}\;{\text{ fraction/Total}}\;{\text{ phenolic }}\;{\text{content}}} \right)\, \times \,{1}00.$$

$${\text{Recovery}}\;{\text{Index}}\left( \% \right)\, = \,{\text{Soluble }}\left( \% \right)\, + \,{\text{Insoluble }}\left( \% \right).$$

### Residual intestinal digesta index

The fraction of the phenolic compounds that are not bioaccessible in the intestine can be calculated according to the equation^30^.

$${\text{RID }}\left( \% \right)\, = \,\left[ {{\text{Intestinal}}\;{\text{insoluble }}\;{\text{fraction/Total }}\;{\text{phenolic}}\;{\text{ content}}} \right]\, \times \,{1}00.$$

### Residual colonic digesta index

The fraction of the phenolic compounds that are not bioaccessible after the colonic fermentation can be calculated according to the equation^30^.

$${\text{RID }}\left( \% \right)\, = \,\left[ {{\text{Colonic}}\;{\text{ insoluble }}\;{\text{fraction/Total}}\;{\text{ phenolic }}\;{\text{content}}} \right]\, \times \,{1}00.$$

### Short Chain fatty acids

One gram of colonic sample was mixed with 5 mL of water and acidified with to a pH of 2.0 using 5 mol/L HCl^30^. The resulting sample mixture was then centrifuged at 10,000 rpm for 10 min at 10 °C. Subsequently, 4 mL of the acid mixture consisting 1% orthophosphoric acid and 1% formic acid was added to the sample mixture. For this study, the standard curves for acetic, propionic, butyric, isobutyric and valeric acids were prepared. A chromatography (7890B Agilent, Santa Clara, USA) equipped with a flame ionization detector (GC-FID), an autosampler (Gilson GX-271, Gilson Inc., Middleton, WI, USA), and an autoinjector was employed to analyze short-chain fatty acids. The capillary column (SGE BP21, 12 × 0.53 nm internal diameter of 0.5 µm film thickness, SGE International, Ringwood, VIC, Australia, P/N 05473) with retention gap kit (including a 2 × 0.53 mm ID guard column, P/N SGE RGK2) was used with an injection volume of 1 µL. Helium served as the carrier gas with a total flow rate of 14.4 mL/min, and the gas consisted of nitrogen, hydrogen, and air with a flow rate of 20, 30, and 300 mL/min, respectively. The oven temperature protocol was as follows: initial 100 °C for 30 s, increase to 180 °C at a rate of 6 °C/min for 1 min, and finally held at 200 °C at a rate of 20 °C /min for 10 min. The detector and injection port temperatures were set at 240 and 200 °C, respectively. Short-chain fatty acids were expressed in mmol/L in this study.

### Statistical analysis

The experimental analyses were conducted in triplicates. Mean differences among different seaweed samples in each digestion section were analyzed by one-way analysis of variance (ANOVA) followed by Tukey’s honestly significant differences (HSD) multiple rank test at *p* ≤ 0.05. ANOVA was carried out via Minitab 19.0 software for windows. Graphs were plotted using GraphPad Prism 9 software, and chemical structures were drawn using BioRender software.

## Data Availability

Data will be made available upon reasonable request to the corresponding author.

## References

[1] Lomartire S, Goncalves AMM (2022). An overview of potential seaweed-derived bioactive compounds for pharmaceutical applications. Mar. Drugs.

[2] Aina O (2022). Seaweed-derived phenolic compounds in growth promotion and stress alleviation in plants. Life (Basel).

[3] Gunathilake T (2022). Seaweed phenolics as natural antioxidants, aquafeed additives, veterinary treatments and cross-linkers for microencapsulation. Mar. Drugs.

[4] Subbiah V, Duan X, Agar OT, Dunshea FR, Barrow CJ, Suleria HAR (2023). Comparative study on the effect of different drying techniques on phenolic compounds in Australian beach-cast brown seaweeds. Algal Res..

[5] Subbiah V, Ebrahimi F, Agar OT, Dunshea FR, Barrow CJ, Suleria HAR (2013). Comparative study on the effect of phenolics and their antioxidant potential of freeze-dried Australian beach-cast seaweed species upon different extraction methodologies. Pharmaceuticals.

[6] Shannon E, Conlon M, Hayes M (2021). Seaweed components as potential modulators of the gut microbiota. Mar. Drugs.

[7] Sensoy I (2021). A review on the food digestion in the digestive tract and the used in vitro models. Curr. Res. Food Sci..

[8] Aleixandre A, Gisbert M, Sineiro J, Moreira R, Rosell CM (2022). In vitro inhibition of starch digestive enzymes by ultrasound-assisted extracted polyphenols from *Ascophyllum nodosum* seaweeds. J. Food Sci..

[9] Mercatante D (2022). Effects of in vitro digestion on the antioxidant activity of three phenolic extracts from olive mill wastewaters. Antioxidants (Basel).

[10] Marques MC, Hacke A, Neto CAC, Mariutti LRB (2021). Impact of phenolic compounds in the digestion and absorption of carotenoids. Curr. Opin. Food Sci..

[11] Loo YT, Howell K, Suleria H, Zhang P, Liu S, Ng K (2023). Fibre fermentation and pig faecal microbiota composition are affected by the interaction between sugarcane fibre and (poly)phenols in vitro. Int. J. Food Sci. Nutr..

[12] Portincasa P (2022). Gut microbiota and short chain fatty acids: Implications in glucose homeostasis. Int. J. Mol. Sci..

[13] Flint HJ (2020). Chapter 6-Variability and Stability of the Human Gut Microbiome.

[14] Charoensiddhi S, Conlon M, Methacanon P, Thayanukul P, Hongsprabhas P, Zhang W (2022). Gut microbiome modulation and gastrointestinal digestibility in vitro of polysaccharide-enriched extracts and seaweeds from *Ulva rigida* and *Gracilaria fisheri*. J. Funct. Foods.

[15] Parkar SG, Trower TM, Stevenson DE (2013). Fecal microbial metabolism of polyphenols and its effects on human gut microbiota. Anaerobe.

[16] Saifullah M, McCullum R, McCluskey A, Vuong Q (2019). Effects of different drying methods on extractable phenolic compounds and antioxidant properties from lemon myrtle dried leaves. Heliyon.

[17] Ma Y, Gao J, Wei Z, Shahidi F (2021). Effect of in vitro digestion on phenolics and antioxidant activity of red and yellow colored pea hulls. Food Chemistry.

[18] Wojtunik-Kulesza K (2020). Influence of in vitro digestion on composition, bioaccessibility and antioxidant activity of food polyphenols—a non-systematic review. Nutrients.

[19] Chandrasekara A, Shahidi F (2012). Bioaccessibility and antioxidant potential of millet grain phenolics as affected by simulated in vitro digestion and microbial fermentation. J. Funct. Foods.

[20] Feng S (2021). Systematic review of phenolic compounds in apple fruits: Compositions, distribution, absorption, metabolism, and processing stability. J. Agric. Food Chem..

[21] Luo J (2022). Bioaccessibility of phenolic compounds from sesame seeds (*Sesamum indicum* L.) during in vitro gastrointestinal digestion and colonic fermentation. J. Food Process. Preserv..

[22] Attri S, Singh N, Singh TR, Goel G (2017). Effect of in vitro gastric and pancreatic digestion on antioxidant potential of fruit juices. Food Biosci..

[23] Wang C (2022). Bioaccessibility and movement of phenolic compounds from tomato (*Solanum lycopersicum*) during in vitro gastrointestinal digestion and colonic fermentation. Food Funct..

[24] Munteanu IG, Apetrei C (2021). Analytical methods used in determining antioxidant activity: A review. Int. J. Mol. Sci..

[25] Qin Y (2018). Release of phenolics compounds from *Rubus idaeus* L. dried fruits and seeds during simulated in vitro digestion and their bio-activities. J. Funct. Foods.

[26] Ma H (2022). Antioxidant activity of vitis davidii foex seed and its effects on gut microbiota during colonic fermentation after in vitro simulated digestion. Foods (Basel, Switzerland).

[27] Attri S, Sharma K, Raigond P, Goel G (2018). Colonic fermentation of polyphenolics from Sea buckthorn (*Hippophae rhamnoides*) berries: Assessment of effects on microbial diversity by Principal Component Analysis. Food Res. Int..

[28] Zhang X-F (2022). Flavonoid constituents of Amomum tsao-ko Crevost et Lemarie and their antioxidant and antidiabetic effects in diabetic rats–in vitro and in vivo studies. Food Funct..

[29] Zhang L (2023). Release of bound polyphenols from wheat bran soluble dietary fiber during simulated gastrointestinal digestion and colonic fermentation in vitro. Food Chem..

[30] Wu H (2022). Bioaccessibility and bioactivities of phenolic compounds from roasted coffee beans during in vitro digestion and colonic fermentation. Food Chem..

[31] Corona G (2016). Gastrointestinal modifications and bioavailability of brown seaweed phlorotannins and effects on inflammatory markers. Br J Nutr.

[32] Hopper W, Mahadevan A (1997). Degradation of catechin by Bradyrhizobium japonicum. Biodegradation.

[33] Hamaker BR, Tuncil YE (2014). A perspective on the complexity of dietary fiber structures and their potential effect on the gut microbiota. J. Mol. Biol..

[34] Garzón AG, Van de Velde F, Drago SR (2020). Gastrointestinal and colonic in vitro bioaccessibility of γ-aminobutiric acid (GABA) and phenolic compounds from novel fermented sorghum food. LWT.

[35] Serra A (2012). Metabolic pathways of the colonic metabolism of flavonoids (flavonols, flavones and flavanones) and phenolic acids. Food Chem..

[36] García-Bofill M (2021). Biocatalytic synthesis of vanillin by an immobilised eugenol oxidase: High biocatalyst yield by enzyme recycling. Appl. Catal. A Gen..

[37] Pheomphun P, Treesubsuntorn C, Thiravetyan P (2019). Effect of exogenous catechin on alleviating O(3) stress: The role of catechin-quinone in lipid peroxidation, salicylic acid, chlorophyll content, and antioxidant enzymes of *Zamioculcas zamiifolia*. Ecotoxicol. Environ. Saf..

[38] Kim H, Kim SY, Sim GY, Ahn JH (2020). Synthesis of 4-hydroxybenzoic acid derivatives in *Escherichia coli*. J. Agric. Food Chem..

[39] Kambourakis S, Draths KM, Frost JW (2000). Synthesis of gallic acid and pyrogallol from glucose: Replacing natural product isolation with microbial catalysis. J. Am. Chem. Soc..

[40] Fernández-Jalao I, Balderas C, Sánchez-Moreno C, De Ancos B (2020). Impact of an in vitro dynamic gastrointestinal digestion on phenolic compounds and antioxidant capacity of apple treated by high-pressure processing. Innov. Food Sci. Emerg. Technol..

[41] He J (2020). Short-chain fatty acids and their association with signalling pathways in inflammation, glucose and lipid metabolism. Int. J. Mol. Sci..

[42] Chambers ES, Preston T, Frost G, Morrison DJ (2018). Role of gut microbiota-generated short-chain fatty acids in metabolic and cardiovascular health. Curr. Nutr. Rep..

[43] Tang S (2021). Effect of Lactobacillus plantarum-fermented mulberry pomace on antioxidant properties and fecal microbial community. LWT.

[44] Li Y, Faden HS, Zhu L (2020). The response of the gut microbiota to dietary changes in the first two years of life. Front. Pharmacol..

[45] Louis P, Flint HJ (2017). Formation of propionate and butyrate by the human colonic microbiota. Environ. Microbiol..

[46] Pylkas AM, Juneja LR, Slavin JL (2005). Comparison of different fibers for in vitro production of short chain fatty acids by intestinal microflora. J. Med. Food.

[47] Badmus UO, Taggart MA, Boyd KG (2019). The effect of different drying methods on certain nutritionally important chemical constituents in edible brown seaweeds. J. Appl. Phycol..

[48] Čagalj M, Skroza D, Tabanelli G, Özogul F, Šimat V (2021). Maximizing the antioxidant capacity of *Padina pavonica* by choosing the right drying and extraction methods. Processes.

[49] Gulsunoglu Z, Karbancioglu-Guler F, Raes K, Kilic-Akyilmaz M (2019). Soluble and insoluble-bound phenolics and antioxidant activity of various industrial plant wastes. Int. J. Food Prop..

[50] Gu C, Suleria HAR, Dunshea FR, Howell K (2020). Dietary lipids influence bioaccessibility of polyphenols from black carrots and affect microbial diversity under simulated gastrointestinal digestion. Antioxidants (Basel).

[51] Mussatto SI, Ballesteros LF, Martins S, Teixeira JA (2011). Extraction of antioxidant phenolic compounds from spent coffee grounds. Sep. Purif. Technol..

[52] Ali A (2021). Comprehensive profiling of most widely used spices for their phenolic compounds through LC-ESI-QTOF-MS(2) and their antioxidant potential. Antioxidants (Basel).

[53] Nebesny E, Budryn G (2003). Antioxidative activity of green and roasted coffee beans as influenced by convection and microwave roasting methods and content of certain compounds. Eur. Food Res. Technol..

[54] Benzie IF, Strain JJ (1996). The ferric reducing ability of plasma (FRAP) as a measure of "antioxidant power": The FRAP assay. Anal. Biochem..

[55] Suleria HA, Barrow CJ, Dunshea FRJF (2020). Screening and characterization of phenolic compounds and their antioxidant capacity in different fruit peels. Foods.

