# Author Correction: In vitro digestion and colonic fermentation of phenolic compounds and their antioxidant potential in Australian beach-cast seaweeds

**DOI:** 10.1038/s41598-024-56269-x

**Published:** 2024-03-06

**Authors:** Vigasini Subbiah, Faezeh Ebrahimi, Osman Tuncay Agar, Frank R. Dunshea, Colin J. Barrow, Hafiz A. R. Suleria

**Affiliations:** 1 Centre for Sustainable Bioproducts, Deakin University, Waurn Ponds, VIC 3217 Australia; 2 School of Agriculture, Food and Ecosystem Sciences, Faculty of Science, The University of Melbourne, Parkville, VIC 3010 Australia

Correction to: *Scientific Reports* 10.1038/s41598-024-54312-5, published online 22 February 2024

The original version of this Article contained errors.

In the Abstract,

“During colonic fermentation, *P. comosa* released the highest levels of phenolic compounds (4.3 mg GAE/g) after 2 h, followed by an increase in flavonoids (0.15 mg QE/g), tannins (0.07 mg CE/g), and antioxidant activity (DPPH: 0.12 mg CE/g; FRAP: 0.61 mg CE/g) after 4 h.”

now reads:

“During colonic fermentation, *P. comosa* released the highest levels of phenolic compounds (4.3 mg GAE/g) after 2 h, followed by an increase in flavonoids (0.15 mg QE/g), tannins (0.07 mg CE/g), and antioxidant activity (DPPH: 0.12 mg TE/g; FRAP: 0.61 mg TE/g) after 4 h.”

In addition, in the Results and discussion section, under the subheading ‘Phenolic and antioxidant activity changes during in vitro digestion’,

“Similarly, in the TCT assay, the tannin content ranged from 0.05 to 0.35 mg CE per g in the undigested phase, 0.063 to 0.352 mg CE per g in the oral phase, 0.036 to 0.474 mg CE per g in the gastric phase and 0.188 to 0.72 CE mg CE per g in the intestinal phase.”

now reads:

“Similarly, in the TCT assay, the tannin content ranged from 0.05 to 0.35 mg CE per g in the undigested phase, 0.063 to 0.352 mg CE per g in the oral phase, 0.036 to 0.474 mg CE per g in the gastric phase and 0.188 to 0.72 mg CE per g in the intestinal phase.”

The original Article has been corrected.

